# The Extract of *Larrea tridentata* Promotes the Synthesis of Silver Nanoparticles and Stimulates Immune Responses in *Penaeus vannamei* Against *Vibrio* spp., Causing Acute Hepatopancreatic Necrosis Disease

**DOI:** 10.3390/microorganisms12112219

**Published:** 2024-11-01

**Authors:** Germán León-Valdez, Wenceslao Valenzuela-Quiñonez, Píndaro Álvarez-Ruiz, Carlos A. Soto-Robles, Eusebio Nava-Perez, Gabriela López-Cervantes, Magnolia Montoya-Mejía

**Affiliations:** 1 Maestría en Recursos Naturales y Medio Ambiente, Instituto Politécnico Nacional-CIIDIR Unidad Sinaloa, Boulevard Juan de Dios Bátiz Paredes 250, Guasave 81101, Sin, Mexico; gleonv2000@alumno.ipn.mx; 2 Departamento de Acuacultura, Instituto Politécnico Nacional-CIIDIR Unidad Sinaloa, Boulevard Juan de Dios Bátiz Paredes 250, Guasave 81101, Sin, Mexico; palvarez@ipn.mx (P.Á.-R.); glopezc@ipn.mx (G.L.-C.); 3 Departamento de Ingeniería Química y Bioquímica, Tecnológico Nacional de México-IT de Los Mochis, Los Mochis 81259, Sin, Mexico; carlos.sr@mochis.tecnm.mx; 4 Departamento de Biotecnología Agrícola, Instituto Politécnico Nacional-CIIDIR Unidad Sinaloa, Boulevard Juan de Dios Bátiz Paredes 250, Guasave 81101, Sin, Mexico; enavap@ipn.mx

**Keywords:** Pacific white shrimp, *Vibrio*, green synthesis, AHPND, MIC

## Abstract

Specific strains of *Vibrio parahaemolyticus* can cause Acute Hepatopancreatic Necrosis Disease (AHPND), a critical issue in shrimp aquaculture despite the application of several control strategies. The use of antibiotics is now restricted due to increasing bacterial resistance and overuse. Silver nanoparticles (AgNPs) have shown potential in shrimp aquaculture, with applications in boosting immunity against certain types of pathogens, promoting growth, and improving survival rates. However, an economically viable solution that protects the organisms has not been found, which is why the search for nanoparticles synthesized with plant extracts is necessary to generate environmentally friendly control strategies. In this study, we synthesized AgNPs from *Larrea tridentata* extract and administered them orally with feed over a 35-day period. Shrimps fed with AgNP-enriched diets showed a significant increase (*p* < 0.05) in mRNA expression of immune-related genes (CTL-5, MNK, SR, and GILT), particularly within the first 24–48 h. No significant differences were observed in growth rates, but survival rates in a challenge against *V. parahaemolyticus* exceeded 85%, higher than the control group. Based on our findings and previous literature, *L. tridentata* can effectively promote the synthesis of AgNPs and shows potential as an antimicrobial agent, without affecting the growth or survival of treated shrimp.

## Simple Summary

Mexico is the world’s sixth-largest producer of farmed white shrimp (*Penaeus vannamei*). Acute Hepatopancreatic Necrosis Disease (AHPND), caused by a strain of *Vibrio parahaemolyticus*, has led to significant economic losses in this industry. Initially, antibiotics were used in feed to control the disease, but this practice has led to environmental changes and increased bacterial resistance and virulence. Silver nanoparticles (AgNPs), generated through green synthesis, offer an alternative treatment that has shown antibacterial properties, destroying the cell membrane or penetrating the cell. In this bioassay, the addition of AgNPs to shrimp feed did not affect survival rates, which were higher than the control group, demonstrating that AgNPs can improve shrimp survival when added to their diet.

## 1. Introduction

Shrimp farming is one of the fastest-growing industries worldwide, *Penaeus vannamei* being one of the most widely cultivated species [1]. However, poor management practices and increasing stocking densities contribute to the incidence and prevalence of infectious diseases, such as Acute Hepatopancreatic Necrosis Disease (AHPND), which can cause high mortality during the first days of cultivation. This disease is characterized by the formation of biofilms on the cuticular surface of the shrimp’s stomach, where toxins are released and subsequently enter the hepatopancreas, causing necrosis that ultimately kills the shrimp [2]. The global spread of multi-drug-resistant pathogens is a major concern, particularly in aquaculture. The excessive use and improper prescription of antibiotics have contributed to the development of bacterial resistance, which now poses a serious threat to both shrimp farming and human health. While *Vibrio parahaemolyticus* has not yet become a direct health risk for humans, shrimp farmers have historically relied on antibiotics without fully considering the environmental repercussions and the potential for bacterial resistance. Therefore, it is imperative to explore alternative solutions that reduce the use of antibiotics and prevent resistance from developing.

Several strategies have been implemented to limit the spread of AHPND, including the use of medicinal plant extracts and nanoparticles [3,4,5]. *Larrea tridentata*, a plant from the Zygophyllaceae family, is endemic to Mexico but can also be found in southern North America [6]. Among the bioactive compounds in this plant, lignans have demonstrated the most promising biological activity, with notable antimicrobial effects against both Gram-positive and Gram-negative bacteria [3].

Silver nanoparticles (AgNPs) are of particular interest due to their properties, including conductivity, antibacterial activity, and biological compatibility [7]. Silver is known to be antagonistic to a broad range of microorganisms, and its most important applications currently lie in the industrial and healthcare sectors [8]. In general, the functional groups in plant extracts serve as reducing agents, reacting with metal ions of the precursor salt to convert them into nanoparticles with varying shapes and sizes [9].

Despite their potential, silver nanoparticles remain underutilized in aquaculture, largely due to a lack of scientific evidence regarding their ion-generating capacity and potential negative effects. Given their significance, this study aims to synthesize silver nanoparticles using *L. tridentata* (Gobernadora) and evaluate their impact on water quality, shrimp survival, and antibacterial activity against *V. parahaemolyticus*, as well as the expression of resistance genes associated with VpAHPND.

## 2. Materials and Methods

### 2.1. Obtaining Plant Material

Vegetative material from “gobernadora” (*L. tridentata*) was collected outside Hermosillo, Sonora, located at 28°51′18.0″ N 111°23′41.2″ W. Subsequently, the leaves were dehydrated on blotting paper and a mash at 27 ± 2 °C for 10 days. The dry samples of each plant were pulverized and sieved using an industrial blender (Thermo Fisher Scientific^®^, Waltham, MA, USA) at 45 °C for 48 h [10]. After the leaves were dried, they were pulverized using a blender until they became a fine powder and stored in a dark container that was completely closed.

### 2.2. Plant Extraction

*L. tridentata* extracts were obtained by mixing 200 mL of distilled water with 40 g of powder sample (20% *w*/*v*) and placed in a bottom flask under constant movement at 24 °C for 1 h. After this time, the solution was filtered through a Whatman No. 1 filter (180 µm thick) and filtered with a Buchner funnel attached to a filter flask.

### 2.3. Green Synthesis

AgNPs were synthesized using the simplified protocol [11], with silver nitrate (AgNO_3_ > 99.8%, Sigma-Aldrich^®^, Burlington, MA, USA) as a precursor, aqueous extracts of *L. tridentata* as a reducing and stabilizer agent, deionized water, and ammonium hydroxide (NH_4_OH) to adjust the pH. To synthesize the high concentration of AgNPs (0.1 M), 140 mL of *L. tridentata* aqueous extract was placed at room temperature in a bottom flask with 70 mL of AgNO_3_ (0.1 M), 35 mL of NH_4_OH, and 455 mL of distilled water. To synthesize the low concentration of AgNPs (0.01 M), we employed 140 mL of *L. tridentata*, 70 mL of AgNO_3_ (0.01 M), and the same volume of NH_4_OH and distilled water. Finally, at both doses, the mixture with a final volume of 700 mL was stirred with a stir plate magnet for at least 4 h to promote nanoparticle synthesis. The samples were stored at room temperature for future use.

### 2.4. Characterization

Sample characterization was carried out with a spectrophotometer (UV–Vis; GENESYS™ 10S, Thermo Fisher Scientific^®^, Waltham, MA, USA) to analyze the surface plasmon resonance using the nanoparticle solution diluted 1:4 with deionized water. The morphological characteristics of the samples were analyzed using transmission electron microscopy (TEM; EDAX, AMETEK Inc.^®^, Devon-Berwyn, PA, USA).

### 2.5. Experimental Animals

Pacific white shrimp (*P. vannamei*) postlarvae 8 ± 0.7 mm were obtained at the “Acuícola 50” shrimp farm, located in El Sacrificio, Guasave, Sinaloa, Mexico (25°27′47.2″ N 108°42′00.8″ W). The specimens were carefully checked to rule out the presence of malformations or signs of disease and were immediately sent to the IPN-CIIDIR where the experiment was carried out. The organisms were placed in a container with seawater saturated with dissolved oxygen, at a salinity of 30 g/L and a temperature of 22 °C for their transportation. Shrimp were maintained at 26 ± 1 °C and fed with a 35% (*w*/*w*) protein commercial diet. Once the acclimatization process was finished, 30 individuals were taken and placed in 200 L capacity tubs before starting the bioassay.

### 2.6. Experimental Diets

For the control diet (CN), 600 g of fine powder was obtained from the commercial feed; grinding was carried out at room temperature (18 °C) with low light intensity, to which 360 mL of distilled water (60%) was added. During the mixing process, 12 g (2% total weight) of sodium alginate (C_6_H_7_NaO_6_) was added, as well as to the rest of the treatments. Subsequently, this mixture was passed to a meat grinder to make the pellets.

Next, 60 mL of the aqueous extract of *L. tridentata* (200 mg/mL) was passed and mixed with 300 mL of distilled water to incorporate into the feed and pelletize the second treatment (Ext). To prepare treatment 3 (lower concentration of nanoparticles) (AgNps-Lt), the same process was carried out, with the difference being that it used 30 mL of the AgNPs 0.01 M silver nanoparticle solution (0.10797 mg/mL Ag) and 330 mL of distilled water to be mixed and taken to the pelletizer. The last treatment (higher concentration of nanoparticles) was carried out in the same way, with the difference being that 300 mL of AgNPs 0.1 M (0.7428 mg/mL Ag) and 60 mL of distilled water were used. The pellets were dried in the laboratory without lighting with a fan for at least 24 h. They were stored in opaque containers with hermetic closure in a freezer at −4 °C, within which the necessary food was taken in weekly rations in covered containers to avoid light exposure.

### 2.7. Vibrio parahaemolyticus Strain

The VpAHPND-E9 strain was obtained from a previously isolated and characterized material, which is in storage at the CIIDIR Unidad Sinaloa facility. The bacterial inoculum was preserved in TSB with 2.5% NaCl and 15% glycerol cryopreserved at −70 °C in 2 at a concentration of 1 × 10^6^ CFU/mL. A vial of 2 mL was taken to culture on TSB agar at room temperature for around 24 h. Colonies were then diluted in sterile saline solution and we measured the optical density at 600 nm (OD600) to subsequently inoculate TCBS agar boxes using various dilutions and observe the number of colonies observed per dilution.

### 2.8. Minimum Inhibitory Concentration (MIC)

MIC is defined as the minimum concentration necessary where bacteria growth is not detected. The MIC against a strain of VpAHPND-E9 (1 × 10^6^ CFU/mL) was determined in a 96-well microplate reader. The culture medium used was TSB using fractional dilutions of silver nanoparticles in concentrations ranging from 297.12 to 0.57 μg/mL. Three replicates per dilution were used and 10 µL of the bacteria was placed in each well. This was grown for 24 h at 30 °C. The presence of color and the absorbance measurement were indicative parameters of bacterial growth. The MIC was taken as the minimum concentration necessary to inhibit the growth of the bacteria.

### 2.9. Experimental Bioassay

Four treatments were established (*n* = 30 shrimp by replicate). Animals were fed with experimental diets for 35 consecutive days; the productive parameters and mortality were recorded daily throughout the experiments. After the bioassay animals were sacrificed, gill samples were withdrawn to determine the expression of immune-relevant genes.

#### Water Parameters

During the experiment, dissolved oxygen concentration (DO, mg/L), temperature (°C), pH, and salinity (g/L) were recorded twice a day (08:00 and 16:00 h). Weekly water samples were taken to determine the concentration of ammonium (NH_4_, mg/mL), nitrites (NO_2_, mg/mL), and nitrates (NO_3_, mg/mL) [12,13]. Also, weekly microbiological analyses were carried out to determine the amount of *Vibrio* spp. and *Bacillus* spp. [14].

### 2.10. VpAHPND-E9 Infection After Experimental Treatment with AgNPs

At the end of the culture, 10 organisms were taken from each treatment of the feeding bioassay. The challenges were carried out in 50 L aquariums with sterile seawater at 25 g/L salinity and with constant aeration at 30 ± 1.0 °C. Infections were carried out by immersion, adding 260,000 CFU/mL of a *V. parahaemolyticus* strain to every experimental unit. After performing the challenge against VpAHPND-E9 and evaluating resistance to the pathogen, survival was recorded at 0, 6, 12, 24, and 48 h post-infection.

### 2.11. Immunological Analysis

Gene expression was calculated by the 2^ΔΔCq^ method [15], using *β*-actin as the reference gene. The stored samples were individually macerated with pistils to later add 500 µL of Trizol. This mixture was centrifuged at 10,000 G at 4 °C for 10 min, and 500 µL was transferred to sterile vials containing 100 µL of chloroform to be centrifuged again at 12,000 G at 4 °C for 15 min to recover the transparent phase and place isopropanol at a 1:1 ratio of the volume obtained (250 µL). This was incubated again and centrifuged according to the mentioned conditions. They were suspended from the vial with 250 µL of 75% ethanol for washing after the last two centrifugations and decanted to obtain a pellet, which was re-suspended in DPC water and placed in a freezer at −80 °C.

#### qPCR Analysis

Real-time PCR (qPCR) cDNA was used to quantify the expression of CTL-3, CTL-5, MNK, SR, ALF, β-actin, and GILT genes. The gene name and reference are indicated in Table 1 and are known to be significantly upregulated by *V. parahaemolyticus* infection. The qPCR was carried out using an Eva Green Bio-Rad^®^ (Hercules, CA, USA). The amplifications [16] were performed in a reaction volume of 15 μL containing 7.5 μL of master PCR mixture, 10 μM of each specific primer (Forward/Reverse), and 5 μL of cDNA dilution. The following steps are included in the amplification program: 95 °C for 5 min followed by 40 cycles of 95 °C for 15 s, 60 °C for 20 s, and 72 °C for 25 s.

### 2.12. Statistical Analysis

Effects of the treatments related to physicochemical and biological parameters were analyzed using a multifactor ANOVA to evaluate the parameters of water quality, growth, and survival of the shrimp. When a significant difference was found, these differences were evaluated with a comparison of means using the Tukey test, with a significance of *p* ≤ 0.05. Gene expression results were analyzed directly using a one-way ANOVA. For these analyses, the IBM SPSS statistics program was used.

## 3. Results and Discussion

### 3.1. Synthesis and Characterization of Silver Nanoparticles

**Figure 1:**
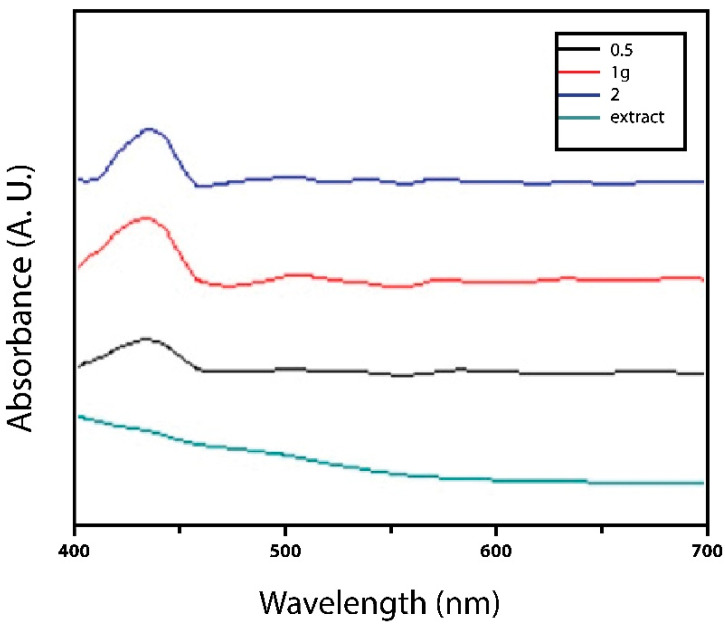
UV–Visible absorbance of silver nanoparticles synthesized from an extract of *L. tridentata* at concentrations 0.5, 1, and 2 g/mL of AgNO_3_ and extract as reducing agent.

The UV–Vis spectra of AgNPs were measured at different concentrations of 0.5–2 g/mL of extract, as shown in Figure 1. All the samples exhibited the peak for AgNPs at 330–450 nm, showing their surface plasmon resonance bands, characteristic of silver nanoparticles; likewise, Córdova-Cisneros et al. obtained nanoparticles of a similar size and shape by synthesizing nanoparticles using aqueous extracts of *L. tridentata* as a precursor, since the metabolites present in the plant promote the synthesis of functional nanoparticles [5]. These HR-TEM results indicate that both treatments worked as expected, showing that *L. tridentata* serves as a reducing and stabilizing agent in the synthesis of Ag nanoparticles.

**Figure 2:**
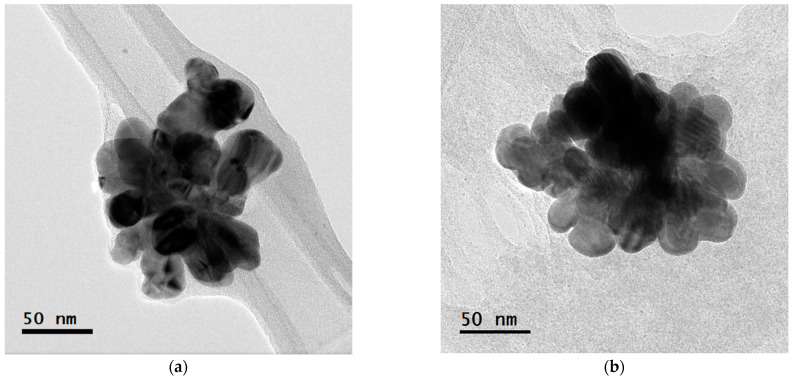
Micrograph of nanoparticles synthesized with *L. tridentata*, spherical in shape with sizes ranging between 20 and 40 nm: (**a**) = AgNPs (-) 0.01 M and (**b**) = AgNPs 0.1 M.

High-resolution transmission electron microscopy (HRTEM) analysis showed that the diameter of the nanoparticles ranged between 20 and 40 nm, with an almost spherical shape, apparently agglomerated (Figure 2).

The synthesized nanoparticles with the highest concentration of silver were agglomerated but maintained the size. Several variables, such as particle size, surface charge, and physicochemical features of water, are related to the effects and transport of the AgNPs and, therefore, their bioavailability and stability [20].

According to Mulenos et al., the changes in shape and agglomeration in nanoparticles are dependent on the surface charge, which determines their antibacterial capacity and their resulting cytotoxicity [21]. These nanoparticles have a diameter between approximately 20 and 40 nm and were suspended in a biological extract derived from *L. tridentata* leaves. These characteristics may have contributed to the nanoparticles’ interaction with the cell walls of *V*. *parahaemolyticus*, allowing their entry into the cytoplasm, which could result in cell death. Moreover, some studies have proposed that the antimicrobial activity of silver nanoparticles can be incited by the adsorption of smaller nanoparticles (<50 nm) on the cell surface, where ions would be released and interact with different types of macromolecules and functional groups present in the cells [22].

The results obtained are similar to those described in the literature; however, using silver nanoparticles synthesized with *L. tridentata* in shrimp farming had not been described up to now, promoting the synthesis of nanoparticles at a lower cost than traditional synthesis, with good antimicrobial capacity and capable of being incorporated to shrimp diets without altering their stability.

### 3.2. Minimum Inhibitory Concentration (MIC)

The concentration of AgNPs that shows a difference in absorbance (Table 2) was established as the MIC. Nanoparticles were able to inhibit the growth of VpAHPND-E9 with an MIC of 21.5 μg/mL Ag (0.01 M), while for the higher concentration of nanoparticles (AgNPs 0.1 M), the minimum dose was 73.8 µg/mL Ag, likely due to its possible agglomeration at high concentrations. In contrast to previous studies, we worked with concentrations of 21.5 μg/mL and 73.8 μg/mL during a period of 35 days with lower mortality than the control group.

*V. parahaemolyticus* was selected for use in this study because it has severely impacted the shrimp industry [23] and can coexist in crustaceans colonizing the digestive tract, gills, and cuticle, producing high mortalities within the first days of shrimp culture [24]. It was found that nanoparticles with low concentrations can have a greater synergistic effect as they are coated with metabolites found in extracts of *Larrea tridentata*, since, in agreement with what was mentioned by other authors, leaves and branches of the plant were used when making the extracts to enhance its antibacterial effect. For example, Vargas-Martinez synthesized silver nanoparticles using *L. tridentata* and found that the MIC was 50 mg/mL of nanoparticles to completely inhibit *Clavibacter michiganensis* [25]. Similarly, Saldívar confirmed that the antimicrobial effect against Gram-positive bacteria is associated with the compounds present in *L. tridentata* [26]. However, it is known that the effect against Gram-negative bacteria may be less effective. These works suggest that its use at low concentrations can inhibit bacteria. It is worth mentioning that in the extracts with dilutions of 5 and 6, there was zero growth. However, at the highest concentration, there was constant growth similar to that of the control group (it was performed several times).

### 3.3. Effects of AgNPs on the Survival of Juvenile White Shrimps

Survival was similar to control (CN) and the AgNP treatments, unlike treatment 2 (commercial feed + *L. tridentata* extract 20%). The growth of the organisms showed no significant differences between weight gain and final weight (Table 3). There are studies that have evaluated the survival and toxicological effects of nanomaterials in aquaculture, particularly metal and metal-oxide NPs [27]. Bhoopathy et al. used chitosan nanoparticles to reinforce the immunity of *L. vannamei* against *V. harvey,* showing an increase in the specific growth rate, daily growth coefficient, and survival rate (*p* < 0.05) [2]. Sharawy et al. achieved a higher survival rate than the negative control by using nanoparticle-based microalgae in shrimp feed [28]. In this study, the addition of AgNPs to the diet improved non-specific immunity, which led to improved survival of individuals exposed to *V. parahaemolyticus* after AgNP applications, in contrast to untreated animals that presented higher mortalities. No clinical signs associated with bioaccumulation by nanoparticles were found; Chávez-Sánchez et al. observed that in a period of 11 days, low concentrations of nanoparticles induced histological damage, obtaining mortality rates of 23% and 20% for the 5.3 and 7.9 μg Ag/μL treatments, respectively [29]. Throughout the bioassay, the addition of nanoparticles to the feed did not interfere with shrimp survival (being the same as in the control), supporting the findings by Juarez-Moreno et al. showing that the use of nanoparticles in feed increases survival without causing bioaccumulation of silver in organisms [30].

### 3.4. Water Quality

**Figure 3:**
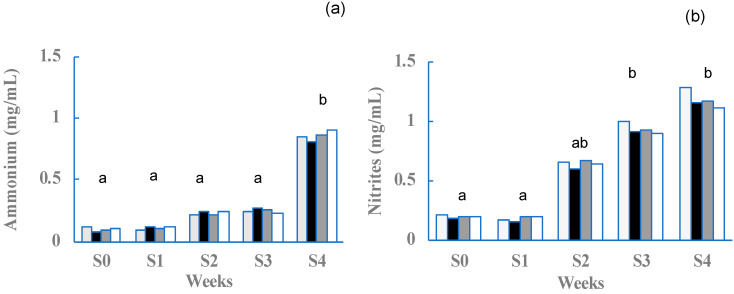
Behavior of nitrogenous compounds throughout the duration of the bioassay. (Light gray) NC = Negative control; (black) Ext = Extract; (dark gray) AgNPs (0.01 M) and (white) AgNPs (0.1 M) silver nanoparticles. (**a**) Ammonium concentration, mg/mL, and (**b**) nitrite concentration, mg/mL. Different letters indicate significant differences between treatments *(p <* 0.05).

Dissolved oxygen (DO), temperature (°C), and pH concentrations were similar between replicates and within appropriate ranges for shrimp farming (Table 4). All conditions remained homogeneous throughout the first 30 days of the bioassay, except for the ammonium and nitrites (Figure 3), which are more toxic to shrimp.

Water quality plays an important role in the stability of nanoparticles, as do the environmental factors to which they are exposed [31]. In aquatic environments, nanoparticles’ efficiency is determined by the phenomenon of aggregation, related to the collision of nanoparticles due to the difference in charges, and is closely related to their size, shape, and composition. It is well known that the higher the salinity, the greater the stability of nanoparticles [32]. No evidence of any particle agglomeration or clinical signs associated with the release of silver ions were found in experimental units.

### 3.5. VpAHPND-E9 Infection After Treatment with AgNPs

**Figure 4:**
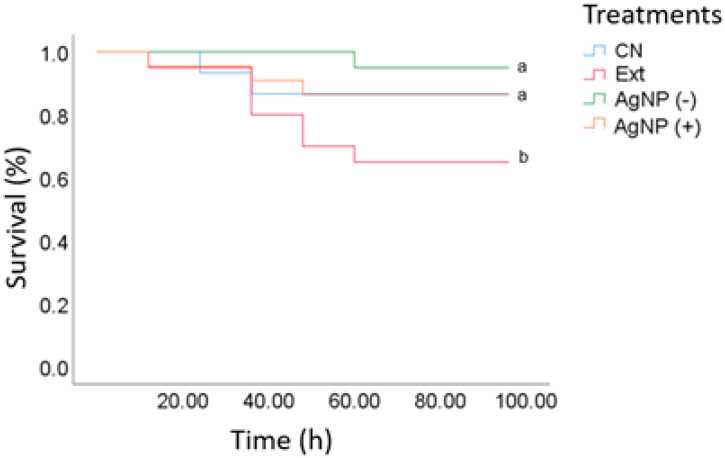
Survival of juvenile white shrimp (*P. vannamei*) challenged with VpAHPND-E9 (260,000 CFU/mL) after being fed experimental diets (CN = Negative Control; Ext = Extract *L. tridentata* 20%; AgNP (-) = low concentration of nanoparticles and AgNP (+) = high concentration of nanoparticles) for a period of 35 days. Different letters indicate significant differences between treatments *(p <* 0.05).

A challenge bioassay was conducted by infecting shrimp with VpAHPND-E9 that were previously fed with nanoparticles, recording mortality every 4 h after the infection (hpi) until the last individual died of the positive control (CN), which was reached at 96 hpi. At 20 hpi, the shrimp did not display discomfort signs, and mortality started at 30 h of infection. Survival rates (95% and 85%) were not significantly different between the low- and high-AgNP diet groups and the positive control (commercial diet) (Figure 4). Organisms infected with VpAHPND-E9 showed survival > 80% for the experimental treatment with *L*. *tridentata* extract. In AHPND-infected shrimp, mortality may reach 100% in the first days of cultivation [33]. Recently, plant-derived compounds and functional feeds against AHPND have been researched, but shrimp mortality was only delayed in time [34,35]. Therefore, it is not clear how shrimp will respond during the critical hours (hpi: 80–100 h). In this study, the application of AgNPs resulted in over 20% mortality during the first 100 hpi, a critical time for shrimp mortality in AHPND, compared to 5% in the positive control group. The most important finding, however, was that the survival curve reached a plateau after 60 h, indicating the end of shrimp mortality. However, the persistence of these effects was not confirmed (Figure 4).

### 3.6. Immune System-Related Genes Expression by qRT-PCR Analysis

**Figure 5:**
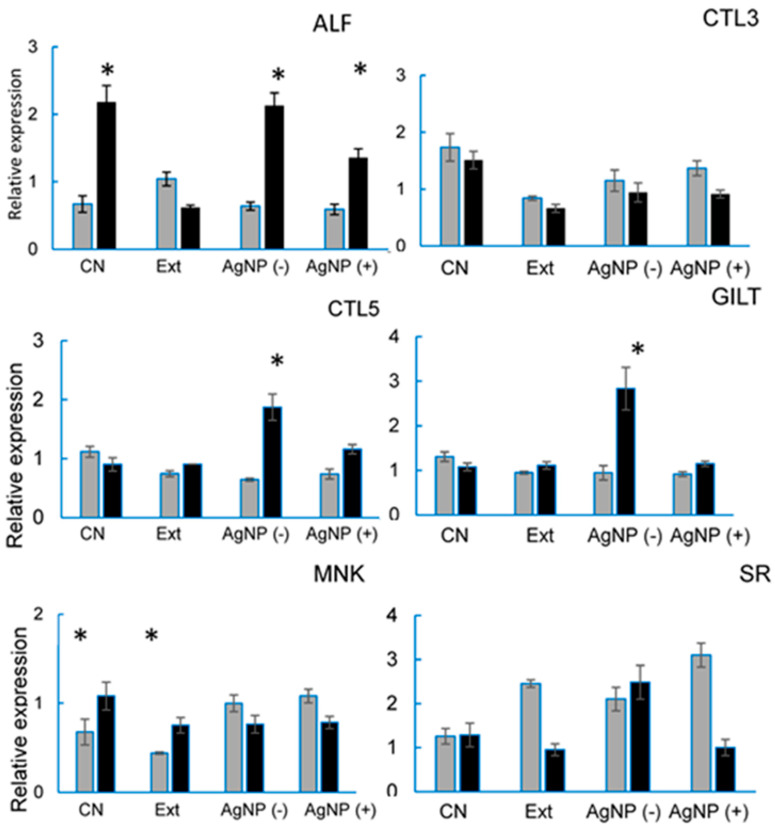
Expression patterns of six genes evaluated in *P. vannamei*. Gray color shows the transcriptional response to being fed with experimental diets for 35 days (T0). In black, the expression of genes related to VpAHPND is observed after being challenged (9 h post-infection). * Significant differences between times post-infection *(p <* 0.05). ALF = Anti-lipopolysaccharide factor-like protein; CTL3 = C-type lectin 3; CTL5 = C-type lectin 5; GILT = Gamma–interferon-inducible lysosomal thiol reductase; MNK = MAP kinase interacting serine; SR = scavenger receptor B1.

All the genes evaluated are related to the immune response against VpAHPND-E9 and different expressions were obtained according to the gene studied (Figure 5). For ALF, treatment 3 (AgNP (-)) obtained similar results to the control treatment (CN); on the other hand, treatment 2 (Ext) had a lower expression. Regarding the CTL-3, treatments with experimental diets showed lower expression than control, although following the same trend. Lectins play essential roles in many biological processes, such as molecular effectors, cell signaling, and pathogen recognition [36]. In this study, feeding shrimp AgNPs did not affect the expression of CTL-3 before VpAHPND infection. In shrimp fed AgNPs daily, the CTL-5 gene was upregulated after infection. This is probably because those lectins have phagocytic capacity [37]; for the MNK, there is no experimental treatment that obtained similar results to the control group. For CTL-5, treatment 3 (AgNP (-)) is observed with greater expression when it has been infected with respect to the other treatments. For SR, basal and post-infection expression were higher in treatment 3 (AgNP (-)). However, in treatments 2 (Ext) and 4 (AgNP (+)), post-infection expression decreased significantly.

Qin et al. showed that the immune response genes expressed in *P. vannamei* were significantly upregulated by *V. parahaemolyticus* infection [17]. The results of this study show that some genes associated with VpAHPND-E9 have lower basal expression, while others, upon infection, exhibit significantly higher expression compared to the control, promoting a positive immune response in at least four of the six genes studied.

Qin et al. showed that the expression of these genes reveals forms of immune response and structural alteration against *V. parahaemolyticus*, demonstrating that they significantly reduce the bacterial elimination ability of *P. vannamei* when the transcriptional response is not adequate [17]; hence, the addition of silver nanoparticles in commercial feed could be used in non-continuous periods to promote immunostimulants without causing bioaccumulation problems and avoid gene overexpression in *P. vannamei* shrimp. The optimal efficiency of AgNPs against *Vibrio parahaemolyticus* can be achieved by optimizing the dose and searching for organic vehicle options for aquaculture pond applications.

## 4. Conclusions

The green synthesis of spherical Ag *nanoparticles* with *L. tridentata* may be effective as a prophylactic method in shrimp aquaculture. The inclusion of AgNPs in feed to enhance survival and prophylaxis against bacteria in infectious challenges did not show any efficacy when compared to the negative control. Nonetheless, at low concentrations, AgNPs demonstrated minimal efficacy in preventing VpAHPND infection. To achieve optimal effectiveness, the concentration must be at least 21.5 µg/mL. Silver nanoparticles promote immune stimulation in shrimp genes (CTL-5, MNK, SR, and GILT) when incorporated into diets or used as an alternative treatment for disease prevention. This study suggests that to reduce the negative effects of VpAHPND, the frequency of administration to *Penaues vannamei* shrimp must be in non-continuous periods to avoid gene overexpression problems. More complex in vivo studies are needed to imply this effectively, in order to determine the best efficiency of AgNPs.

## Figures and Tables

**Table 1:** Primers used to amplify specific sequences bound to genes related to immunity against *V. parahaemolyticus* in *P. vannamei*.

Gen	Name	Primer	Sequence	Cite
ALF	Anti-lipopolysaccharide factor-like protein	qALFP-F	CGAATCTGCGAACTCCAT	[17] 2018
		qALFP-R	GAATAAGAACAGTAGTGACCC	
CTL-3	C-type lectin 3	qCTL3-F	AAACCCTGGATTCGTCAA	[17] 2018
		qCTL3-R	AAACCTTAGCTTAGAGTGGC	
CTL-5	C-type lectin 5	qCTL5-F	TGGCTTCTGTCAGGGTTTCC	[18] 2019
		qCTL5-R	CGTCCGTCCACACGAACTC	
GILT	Gamma-interferon-inducible lysosomal thiol reductase	qGILT-F	GAGTGCAAGGGCAACATG	[17] 2018
		qGILT-R	GGAACGAAGTAGAGGGAAGG	
MNK	MAP kinase interacting serine	qMNK-F	AGCATGAACCAGGATGAGG	[17] 2018
		qMNK-R	AGCCTGTGGCAGTAACGAG	
SR	scavenger receptor B1	qSR-F	GCTGGTGGAGATGTGGTTC	[17] 2018
		qSR-R	TGGTGTTGTTCTTCGGGTA	
β-actin	Beta actine	qLvActin-F	CCACGAGACCACCTACAAC	[19] 2007
		qLvActin-R	AGCGAGGGCAGTGATTTC	

**Table 2:** Experimental dilutions of extracts of *Larrea tridentata* and Ag nanoparticle treatments and minimum inhibitory concentrations (MIC) calculated against *Vibrio parahaemolyticus* (VpAHPND).

Dilution	Extracts (mg/mL)	AgNPs (0.01 M) (μg/mL)	AgNPs (0.1 M) (μg/mL)
1	79.5	42.9	295.2
2	39.9	21.5	147.6
3	19.9	10.7	73.8
4	9.9	5.4	36.9
5	4.9	2.6	18.2
6	2.5	1.4	9.3
7	1.1	0.6	3.9
MIC	10–20 μg/mL	21.5 μg/mL	73.8 μg/mL

**Table 3:** Productive parameters of white shrimp *P. vannamei* fed with experimental diets added with nanoparticles of Ag (AgNPs) for 4 weeks.

Treatments	Initial Weight	Final Weight	Weight Gain (g)	Survival (%)
NC	1.86	4.56	2.64	82 ^a^
EXT	1.81	4.52	2.53	55 ^b^
AgNPs (0.01 M)	1.77	4.43	2.25	82 ^a^
AgNPs (0.1 M)	1.71	4.44	2.74	74 ^a^

The treatments were: 1, Negative Control (NC); 2, Extract *L. tridentata* 20% (Ext); 3, low concentration of nanoparticles (AgNPs = 0.10797 mg/mL of Ag); 4, high concentration of nanoparticles (AgNPs = 0.7428 mg/mL of Ag). Different superscripts are significantly different (*p* < 0.05).

**Table 4:** Physicochemical parameters of water control during experimental bioassay with nanoparticles of Ag (AgNPs) for 4 weeks.

Parameter	NC	Extracts	AgNPs (0.01 M)	AgNPs (0.1 M)
DO (mg/L)	3.8 ± 0.1	3.8 ± 0.1	3.8 ± 0.1	3.8 ± 0.1
Temperature (°C)	27 ± 0.5	27 ± 0.5	27 ± 0.1	27 ± 0.5
pH	7.9 ± 0.1	7.9 ± 0.1	7.9 ± 0.1	7.9 ± 0.1
Salinity (g/L)	26 ± 0.1	26 ± 0.1	26 ± 0.1	26 ± 0.1
NO_2_^−^ (mg/L)	0.66 ± 0.3 ^a^	0.6 ± 0.3 ^a^	0.49 ± 0.3 ^b^	0.61 ± 0.3 ^a^
NO_3_ (mg/L)	5.2± 0.4	4.9 ± 0.4	5.1 ± 0.4	5.2 ± 0.4
NH_4_ (mg/L)	0.8 ± 0.1	0.8 ± 0.1	0.8 ± 0.1	0.8 ± 0.1
*Vibrio* spp. (CFU/mL) 10^2^	104 ^a^	85 ^b^	132 ^a^	150 ^ac^
*Bacillus* spp. (CFU/mL) 10^2^	4	6	5	4

The treatments were: 1, Negative Control (NC); 2, Extract *L. tridentata* 20% (Ext); 3, low concentration of nanoparticles (AgNPs = 0.10797 mg/mL of Ag); 4, high concentration of nanoparticles (AgNPs = 0.7428 mg/mL of Ag). Different superscripts are significantly different (*p* < 0.05).

## Data Availability

The data that support the findings of this study are available on request from the corresponding author.
